# Delivering progranulin to neuronal lysosomes protects against excitotoxicity

**DOI:** 10.1016/j.jbc.2021.100993

**Published:** 2021-07-21

**Authors:** Skylar E. Davis, Jonathan R. Roth, Qays Aljabi, Ahmad R. Hakim, Katherine E. Savell, Jeremy J. Day, Andrew E. Arrant

**Affiliations:** 1 Department of Neurology, Center for Neurodegeneration and Experimental Therapeutics, University of Alabama at Birmingham, Birmingham, Alabama, USA; 2 Alzheimer’s Disease Center, Evelyn F. McKnight Brain Institute, University of Alabama at Birmingham, Birmingham, Alabama, USA; 3 Department of Neurobiology, University of Alabama at Birmingham, Birmingham, Alabama, USA

**Keywords:** progranulin, lysosome, excitotoxicity, autophagy, cell death, protein secretion, neurodegenerative disease, frontotemporal dementia, 4-MU, 4-methylumbelliferone, AD, Alzheimer’s disease, FTD, frontotemporal dementia, L-PGRN, lysosome-targeted progranulin, LDH, lactate dehydrogenase, MEA, multielectrode array, MTT, methylthiazolyldiphenyl-tetrazolium bromide, NCL, neuronal ceroid lipofuscinosis, PGRN, progranulin

## Abstract

Loss-of-function mutations in progranulin (*GRN*) are a major genetic cause of frontotemporal dementia (FTD), possibly due to loss of progranulin’s neurotrophic and anti-inflammatory effects. Progranulin promotes neuronal growth and protects against excitotoxicity and other forms of injury. It is unclear if these neurotrophic effects are mediated through cellular signaling or through promotion of lysosomal function. Progranulin is a secreted proprotein that may activate neurotrophic signaling through cell-surface receptors. However, progranulin is efficiently trafficked to lysosomes and is necessary for maintaining lysosomal function. To determine which of these mechanisms mediates progranulin’s protection against excitotoxicity, we generated lentiviral vectors expressing progranulin (PGRN) or lysosome-targeted progranulin (L-PGRN). L-PGRN was generated by fusing the LAMP-1 transmembrane and cytosolic domains to the C-terminus of progranulin. L-PGRN exhibited no detectable secretion, but was delivered to lysosomes and processed into granulins. PGRN and L-PGRN protected against NMDA excitotoxicity in rat primary cortical neurons, but L-PGRN had more consistent protective effects than PGRN. L-PGRN’s protective effects were likely mediated through the autophagy-lysosomal pathway. In control neurons, an excitotoxic dose of NMDA stimulated autophagy, and inhibiting autophagy with 3-methyladenine reduced excitotoxic cell death. L-PGRN blunted the autophagic response to NMDA and occluded the protective effect of 3-methyladenine. This was not due to a general impairment of autophagy, as L-PGRN increased basal autophagy and did not alter autophagy after nutrient starvation. These data show that progranulin’s protection against excitotoxicity does not require extracellular progranulin, but is mediated through lysosomes, providing a mechanistic link between progranulin’s lysosomal and neurotrophic effects.

Loss-of-function mutations in progranulin (*GRN*) are one of the most common genetic causes of frontotemporal dementia (FTD) (1, 2). The majority of these mutations cause progranulin haploinsufficiency, with *GRN* mutation carriers having less than 50% of normal circulating progranulin levels (3, 4). The presence of loss-of-function mutations on both *GRN* alleles, resulting in nearly complete progranulin deficiency, typically causes the lysosomal storage disorder neuronal ceroid lipofuscinosis (NCL) (5, 6, 7). The *GRN* rs5848 polymorphism is associated with mild progranulin insufficiency and with increased risk for both FTD and Alzheimer’s disease (AD) (8, 9). These data show a dose-dependent relationship of progranulin insufficiency with neurodegenerative disease.

While progranulin has diverse effects on many cell types throughout the body (10, 11, 12, 13, 14), loss of its neurotrophic and anti-inflammatory effects may be key for the association of progranulin insufficiency with neurodegenerative disease. Progranulin enhances neuronal survival (15, 16, 17), increases growth of axons and dendrites (15, 18, 19, 20), promotes axonal regrowth after injury (21, 22), and protects neurons from death due to hypoxia, oxidative stress, and excitotoxicity (23, 24, 25). *Grn*^−/−^ neurons exhibit signs of autophagy-lysosomal dysfunction (26), have impaired dendritic growth (19), are slower to recover from injury (21, 22, 27), and are more vulnerable to TDP-43 mislocalization and aggregation (26, 28, 29). Progranulin also regulates inflammatory responses to injury (30, 31) and restrains inflammation in macrophages and microglia (32, 33). *Grn*^−/−^ microglia exhibit signs of lysosomal dysfunction (34), secrete high levels of inflammatory cytokines (33), and have deficits in phagocytosis (35) and motility (36).

The mechanisms underlying progranulin’s neurotrophic and anti-inflammatory effects are unclear and may involve some combination of extracellular signaling and promotion of lysosomal function. A large portion of newly synthesized progranulin is secreted through the Golgi secretory pathway (37, 38). While in the extracellular space, progranulin can interact with several signaling receptors, including EphA2 (39), Notch (27), and possibly Tnf receptors (40, 41). However, extracellular progranulin is efficiently taken up by many cell types, including neurons, and trafficked to lysosomes (42, 43, 44, 45). Progranulin is necessary for maintaining lysosomal function, as shown by the development of NCL in homozygous *GRN* mutation carriers (5, 6, 7). While the function of progranulin in lysosomes is not well understood, progranulin facilitates the activity of several lysosomal enzymes (22, 46, 47, 48, 49, 50, 51, 52, 53), including the critical protease cathepsin D (22, 46, 47, 52, 53).

An important factor in progranulin’s function is its proteolytic cleavage into granulins, which are also bioactive (15, 22, 30, 53, 54, 55, 56). Progranulin contains one partial and seven full granulin domains and can be cleaved into granulins by extracellular and lysosomal proteases (57, 58, 59, 60, 61). Progranulin is rapidly cleaved into granulins in lysosomes (43), and granulins may mediate some of progranulin’s effects on lysosomal enzymes (22, 52, 53). Granulins may also mediate some of progranulin’s neurotrophic effects (15, 54). In other cases, progranulin and granulins may exert opposing effects (30, 55, 56).

The objective of this study was to investigate the mechanisms underlying progranulin’s neurotrophic effects, with a focus on progranulin’s protective effects against excitotoxicity. Addition of recombinant progranulin to neuronal culture media activates signaling pathways associated with neurotrophic receptors and protects against hypoxia, oxidative stress, and excitotoxicity (23, 24, 25). While this might suggest extracellular signaling by progranulin, progranulin added to cell culture media is rapidly taken up and trafficked to lysosomes (42, 43, 45), making it difficult to interpret these findings.

To determine whether progranulin exerts neuroprotective effects through extracellular signaling or by acting in lysosomes, we generated lentiviral constructs expressing human progranulin (PGRN) or lysosome-targeted human progranulin (L-PGRN). Lysosomal targeting was achieved by fusion of the transmembrane and cytosolic domains of LAMP-1 to the C-terminus of progranulin, a strategy that has been employed in the study of other lysosomal proteins (62). L-PGRN maintained the lysosomal localization of progranulin, but was not secreted, enabling investigation of progranulin’s lysosomal effects without the potential for extracellular signaling. We tested the protective effects of PGRN and L-PGRN against NMDA excitotoxicity in primary cortical neurons and found that L-PGRN exhibited even more consistent protective effects than PGRN. Further investigation indicated that these protective effects were mediated *via* the autophagy-lysosomal pathway.

## Results

### Generation of lysosome-targeted progranulin

**Figure 1:**
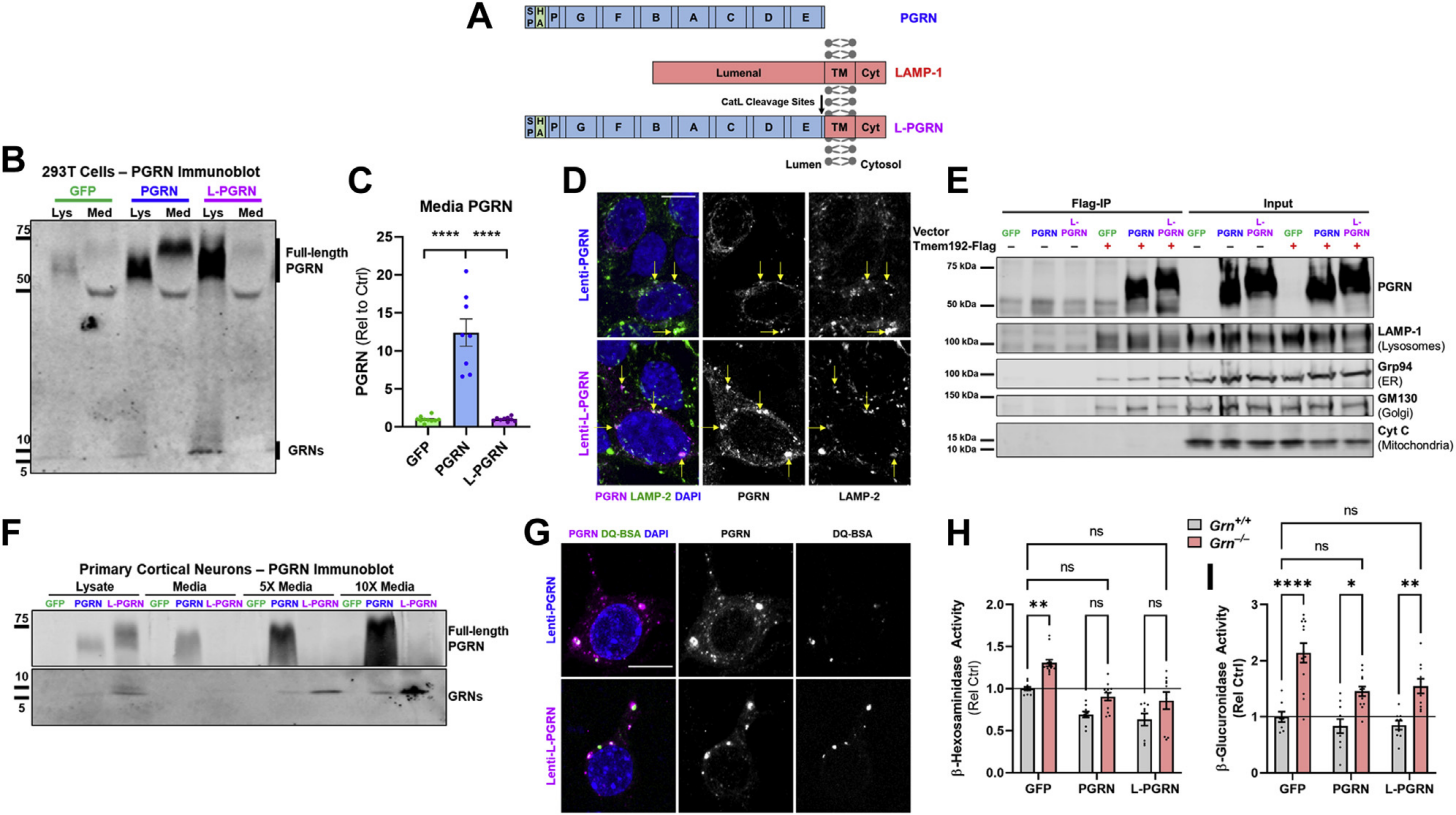
**Generation and characterization of a lysosome-targeted progranulin vector.***A*, lysosome-targeted progranulin (L-PGRN) was generated by fusing the transmembrane domain and cytosolic tail of LAMP-1 to the C-terminus of progranulin. The PGRN and L-PGRN vectors used in this study had an HA tag inserted after the signal peptide. *B*, transduction of HEK293T cells with a lentiviral vector expressing PGRN under the PGK promoter increased progranulin levels in both cell lysates and conditioned media. In contrast, transduction with an L-PGRN vector increased progranulin only in cell lysates and failed to increase progranulin levels above baseline in conditioned media (*C*, ANOVA effect of vector, *p* < 0.0001, ∗∗∗∗*p* < 0.0001 by Tukey’s post-hoc test, n = 8–10 per group). Both vectors also increased granulins in cell lysates, with L-PGRN producing higher levels of granulins than PGRN. *D*, progranulin expressed by both the PGRN and L-PGRN vectors localized to LAMP-2 positive vesicles (*yellow arrows*). *E*, additionally, lysosomal enrichment by immunoprecipitation of Tmem192-Flag (63) supported lysosomal localization of progranulin expressed by the PGRN and L-PGRN vectors. Immunoprecipitates were probed for progranulin and markers of lysosomes (LAMP-1), endoplasmic reticulum (ER) (Grp94), Golgi (GM130), and mitochondria (Cytochrome C (Cyt C)). *F*, similarly, transduction of rat primary neurons with lenti-PGK-PGRN increased progranulin in both lysates and conditioned media, while transduction with lenti-PGK-L-PGRN only increased progranulin levels in cell lysates. Both vectors increased granulins in cell lysates and in conditioned media, with L-PGRN producing higher levels of granulins in both compartments. *G*, immunostaining of neurons transduced with each vector showed robust colocalization of progranulin immunoreactivity with lysosomes labeled with DQ-BSA. *H* and *I*, transduction with both PGRN and L-PGRN at least partially normalized the elevated lysosomal enzyme activity of *Grn*^−/−^ primary cortical neurons, indicated that L-PGRN retains the functional effects of PGRN in lysosomes (*H*, β-hexosaminidase, ANOVA effect of genotype, *p* < 0.0001, effect of vector, *p* < 0.0001, *I*, β-glucuronidase, ANOVA effect of genotype, *p* < 0.0001, effect of vector, *p* = 0.003. ∗*p* < 0.05, ∗∗*p* < 0.01, ∗∗∗∗*p* < 0.0001 by Tukey’s post-hoc test, n = 6–13 per group. Neurons were transduced shortly after plating and enzyme activity was assessed at DIV14). Representative 60× images of progranulin/LAMP-2 or progranulin/DQ-BSA are shown in *D* and *G* with 10 μm scale bars. Cyt, cytosolic domain; TM, transmembrane domain.

To deliver progranulin to lysosomes while bypassing its typical secretion, we generated a lentiviral construct expressing lysosome-targeted progranulin (L-PGRN). L-PGRN was generated by fusing the transmembrane domain and cytosolic tail of LAMP-1 to the C-terminus of progranulin (Fig. 1*A*) (62). Initial analysis in 293T cells showed that transduction with a lentiviral vector expressing L-PGRN under the PGK promoter did not produce any detectable change in extracellular progranulin levels (Fig. 1, *B* and *C*), while transduction with a PGRN lentiviral vector dramatically increased extracellular progranulin. L-PGRN was cleaved into granulins (Fig. 1*B*) and generated even higher levels of granulins than PGRN, consistent with lysosomal delivery of virally expressed L-PGRN without loss to secretion. Coimmunostaining of progranulin and LAMP-2 (Fig. 1*D*) supported localization of both PGRN and L-PGRN to late-endosomes/lysosomes. Enrichment of lysosomes using Tmem192-Flag immunoprecipitation (63) (Fig. 1*E*) also indicated that L-PGRN maintained the typical lysosomal localization of progranulin. These Tmem192 immunoprecipitates contained low levels of endoplasmic reticulum (Grp94) and Golgi (GM130) markers (Fig. 1*E*), which may have been due to either longer immunoprecipitation or use of a different cell type (293T) than the initial study of Tmem192 immunoprecipitation in HeLa cells (63). However, these immunoprecipitates still appeared to be enriched with lysosomes based on high levels of LAMP-1 (Fig. 1*E*) and LAMP-2 (not shown).

Similar results were obtained in primary cortical neurons, in which L-PGRN did not produce detectable levels of extracellular progranulin, even when media was concentrated tenfold (Fig. 1*F*). In contrast to 293T cells, L-PGRN increased levels of granulins in conditioned media of primary neurons, which might be due to lysosomal exocytosis. Colocalization of progranulin with DQ-BSA confirmed that L-PGRN was delivered to lysosomes (Fig. 1*G*).

### Progranulin and lysosome-targeted progranulin normalize lysosomal enzyme activity in progranulin-deficient neurons

Having found that L-PGRN is delivered to lysosomes and cleaved into granulins without being secreted, we next tested whether L-PGRN retains progranulin’s functional effects in lysosomes. Similar to brain tissue of *Grn*^−/−^mice (64, 65, 66, 67), *Grn*^−/−^ primary cortical neurons exhibit elevated activity of lysosomal enzymes such as β-hexosaminidase (Fig. 1*H*) and β-glucuronidase (Fig. 1*I*). Transduction with both PGRN and L-PGRN lentiviral vectors at least partially normalized activity of both enzymes, indicating that L-PGRN retains the lysosomal function of PGRN.

### Progranulin and lysosome-targeted progranulin protect cortical neurons against NMDA excitotoxicity

**Figure 2:**
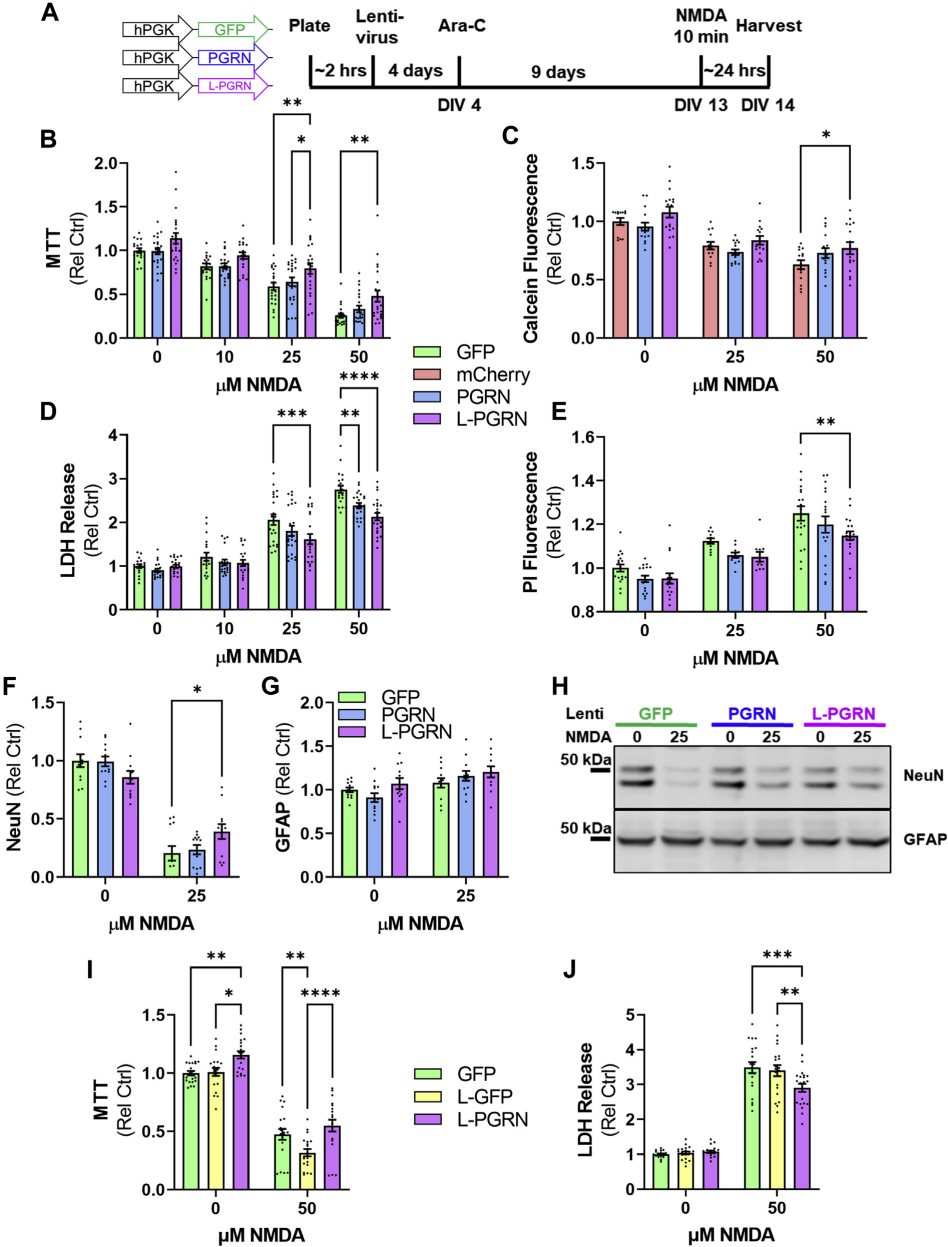
**Extracellular progranulin is not required to protect against NMDA excitotoxicity.***A*, primary neuronal cultures were transduced with vectors expressing GFP, mCherry, PGRN, or L-PGRN under the PGK promoter shortly after plating. At DIV13, the neurons were exposed to 10, 25, or 50 μM NMDA for 10 min. Twenty-four hours later, neurons were analyzed for markers of viability (*B* and *C*) and cell death (*D* and *E*). L-PGRN protected neurons from loss of viability after NMDA treatment in both the MTT (*B*, ANOVA effect of NMDA *p* < 0.0001, effect of vector, *p* < 0.0001, n = 18–23 per group) and calcein (*C*, ANOVA effect of NMDA *p* < 0.0001, effect of vector, *p* = 0.0062, n = 13–17 per group) assays. Both PGRN and L-PGRN protected against cell death as measured by LDH release (*D*, ANOVA effect of NMDA *p* < 0.0001, effect of vector, *p* < 0.0001, NMDA x vector interaction, *p* = 0.0147, n = 17–23 per group), and L-PGRN also reduced cell death as measured by propidium iodide fluorescence (*E*, ANOVA effect of NMDA *p* < 0.0001, effect of vector, *p* = 0.0024, n = 10–19 per group). *F* and *H*, L-PGRN also protected against neuronal loss as measured by loss of NeuN immunoreactivity after 25 μM NMDA treatment (ANOVA effect of NMDA *p* < 0.0001, NMDA x vector interaction, *p* = 0.0056, n = 12 per group), but did not alter levels of the astrocytic protein GFAP (*G* and *H*). *I* and *J*, to control for potential nonspecific effects of increased delivery of protein to lysosomes, neurons were transduced with a PGK lentiviral vector expressing lysosome-targeted GFP (L-GFP) prior to treatment with 50 μM NMDA as described in *A*. In contrast to L-PGRN, L-GFP worsened the loss of viability in the MTT assay (*I*, ANOVA effect of NMDA *p* < 0.0001, effect of vector *p* < 0.0001, n = 20 per group) and failed to protect against cell death as assessed by LDH release (*J*, ANOVA effect of NMDA *p* < 0.0001, effect of vector *p* = 0.0357, NMDA x vector interaction *p* = 0.0060, n = 20 per group). For all analyses ∗*p* < 0.05, ∗∗*p* < 0.01, ∗∗∗*p* < 0.001, and ∗∗∗∗*p* < 0.0001 by Tukey’s post-hoc test.

We next investigated whether L-PGRN retained progranulin’s neuroprotective effects (15, 16, 17, 21, 23, 25, 27), which have been proposed to be mediated either by neurotrophic signaling (25, 27) or enhancement of lysosomal function (21, 22). Progranulin reduces NMDA excitotoxicity in primary cortical neurons (25), so we tested whether L-PGRN could exert similar effects using assays for cell viability (MTT (methylthiazolyldiphenyl-tetrazolium bromide) and calcein) and cell death (LDH (lactate dehydrogenase) release and propidium iodide staining). Neurons were transduced shortly after plating with lentiviral vectors expressing GFP, PGRN, or L-PGRN under the PGK promoter (Fig. 2*A*). For the calcein assay, which generates green fluorescence in living cells, an mCherry lentiviral vector was used as a control. At DIV13, neurons were treated with NMDA for 10 min, then analyzed for viability and cell death 24 h later. Consistent with a prior report (25), PGRN protected neurons against cell death as assessed by LDH release (Fig. 2*D*), though it did not exhibit statistically significant effects in the other assays. In contrast, L-PGRN exhibited protective effects relative to GFP across all assays, preserving neuronal viability (Fig. 2, *B* and *C*) and reducing neuronal death (Fig. 2, *D* and *E*). L-PGRN was more protective than PGRN only in the MTT assay, as PGRN exhibited statistically nonsignificant trends for protection in the calcein (Fig. 2*C*) and propidium iodide (Fig. 2*E*) assays. To confirm that L-PGRN’s protective effects in these assays reflected enhanced neuronal survival after NMDA treatment, we measured levels of NeuN, a neuronal marker, and GFAP, an astrocytic marker, in our cultures 24 h after NMDA treatment. L-PGRN protected cultures against loss of NeuN (Fig. 2, *F* and *H*), but did not alter GFAP levels (Fig. 2, *G* and *H*).

Since L-PGRN had more consistent protective effects than PGRN, we tested whether these effects might be due to a nonspecific effect of enhanced protein delivery to lysosomes by comparing L-PGRN to lysosome-targeted GFP (L-GFP) (Fig. S1). Like L-PGRN, L-GFP was generated by fusing the transmembrane domain and cytosolic tail of LAMP-1 to the C-terminus of GFP. Unlike L-PGRN, L-GFP did not protect against NMDA excitotoxicity (Fig. 2, *I* and *J*), indicating that L-PGRN’s protective effects are likely mediated by progranulin’s actions in lysosomes. The broader protective effects of L-PGRN than PGRN are consistent with this conclusion, as transduction with the L-PGRN vector delivers higher levels of intracellular progranulin (Fig. 1, *B* and *F*) than the PGRN vector.

### Conditioned media does not confer the full neuroprotective effects of lysosome-targeted progranulin

**Figure 3:**
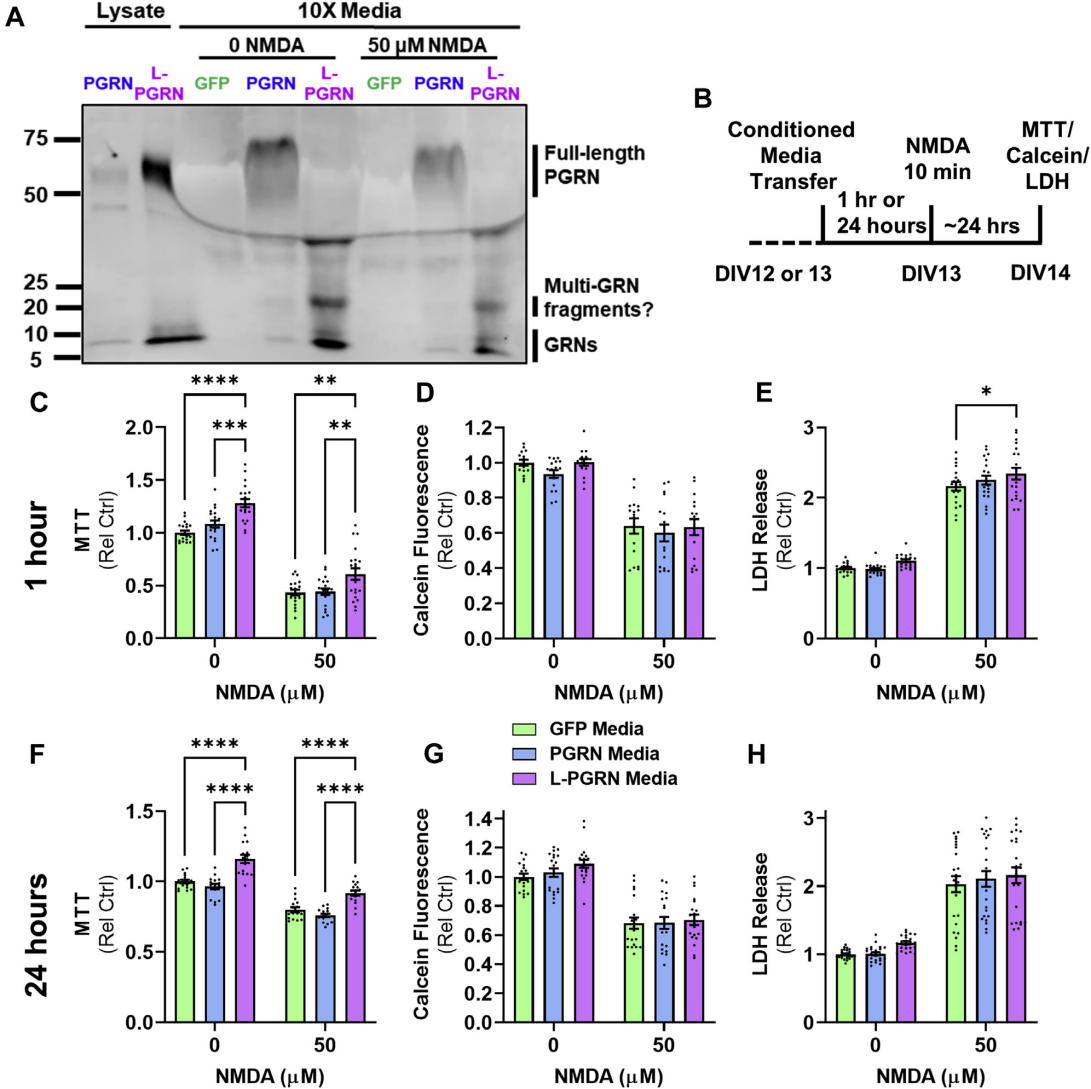
**Conditioned media from L-PGRN–transduced neurons does not confer the full protective effects of transduction with L-PGRN.***A*, analysis of conditioned media from primary neurons treated with 0 or 50 μM NMDA revealed that both the PGRN and L-PGRN vectors increased levels of fully cleaved granulins (5–10 kDa) and potential multigranulin fragments (20–25 kDa) in cellular lysates and conditioned media. Extracellular progranulin was not detectable in conditioned media from L-PGRN–transduced neurons under either condition, even when media was concentrated 10-fold. *B*, to determine if extracellular granulins might mediate the protective effects of L-PGRN against NMDA excitotoxicity, we pretreated untransduced neurons with conditioned media from GFP-, PGRN, or L-PGRN–transduced neurons either 1 (*C*–*E*) or 24 (*F*–*H*) hours before applying NMDA for 10 min and assessing viability (MTT and calcein assays) or cell death (LDH assay). L-PGRN conditioned media increased viability both at baseline and after NMDA treatment with both 1 h (*C*, ANOVA effect of NMDA, *p* < 0.001, effect of media, *p* < 0.0001, n = 19–20 per group) and 24 h (*F*, ANOVA effect of NMDA, *p* < 0.001, effect of media, *p* < 0.0001, n = 16 per group) of pretreatment. However, L-PGRN conditioned media did not protect against loss of viability in the calcein assay at either duration of pretreatment (*D* and *G*). Assessment of LDH release revealed that L-PGRN conditioned media worsened cell death relative to GFP conditioned media after 1 h of pretreatment (*D*, ANOVA effect of NMDA, *p* < 0.001, effect of media, *p* = 0.0201, n = 19–20 per group), with a similar trend after a 24-h pretreatment (*G*, ANOVA effect of NMDA, *p* < 0.001, n = 24 per group). For all analyses ∗*p* < 0.05, ∗∗*p* < 0.01, and ∗∗∗∗*p* < 0.0001 by Tukey’s post-hoc test.

Our findings to this point indicated that extracellular progranulin is not necessary for protecting against excitotoxicity. In support of this possibility, we were unable to detect full-length extracellular progranulin in tenfold concentrated conditioned media from L-PGRN–transduced neurons 24 h after NMDA treatment (Fig. 3*A*), showing that progranulin did not leak out of dying neurons at detectable levels. However, conditioned media from L-PGRN–transduced neurons contained fully-cleaved granulins (5–10 kDa) and possible multigranulin fragments resulting from partially cleaved progranulin (20–25 kDa) both at baseline and after NMDA treatment (Fig. 3*A*), raising the possibility that extracellular granulins or multigranulin fragments might mediate L-PGRN’s protective effects.

To determine if extracellular granulins could mediate L-PGRN’s neuroprotective effects, we tested whether PGRN or L-PGRN conditioned media could mimic the protective effects of transduction with PGRN or L-PGRN lentiviral vectors. We transferred media from GFP, PGRN, or L-PGRN–transduced neurons to naïve primary cortical neurons 1 h before treatment with NMDA (Fig. 3*B*), a time course in which treatment with recombinant progranulin exerts protective effects (25). We observed no effects of PGRN conditioned media, which may be due to the presence of lower levels of progranulin than were used in prior studies reporting protective effects of recombinant progranulin in neuronal culture media (23, 24, 25). In contrast, L-PGRN conditioned media increased viability in the MTT assay both at baseline and after NMDA treatment (Fig. 3*C*). However, neurons treated with L-PGRN media had a comparable drop in viability in the calcein assay as neurons treated with GFP media (Fig. 3*D*), and higher levels of LDH release (Fig. 3*E*), indicating more cell death.

Since a 1-h pretreatment with L-PGRN conditioned media increased viability in the MTT assay, we tested whether longer pretreatment would provide the full protective effects of L-PGRN. We transferred GFP, PGRN, and L-PGRN conditioned media to naïve neurons 24 h before treatment with NMDA (Fig. 3*B*). This longer pretreatment with L-PGRN conditioned media produced similar results as the 1-h pretreatment, with increased viability in the MTT assay (Fig. 3*F*), but no change from GFP in either the calcein assay (Fig. 3*G*) or LDH release (Fig. 3*H*). Since the increase in MTT viability was not replicated in either the calcein or LDH assays, this effect may reflect changes in cellular mitochondrial activity rather than an increased number of living cells. These data show that extracellular granulins and multigranulin fragments do not replicate the full protective effect of transduction with L-PGRN in a time frame consistent with acute cellular signaling.

### Selective transduction of neurons with lysosome-targeted progranulin protects against NMDA excitotoxicity

**Figure 4:**
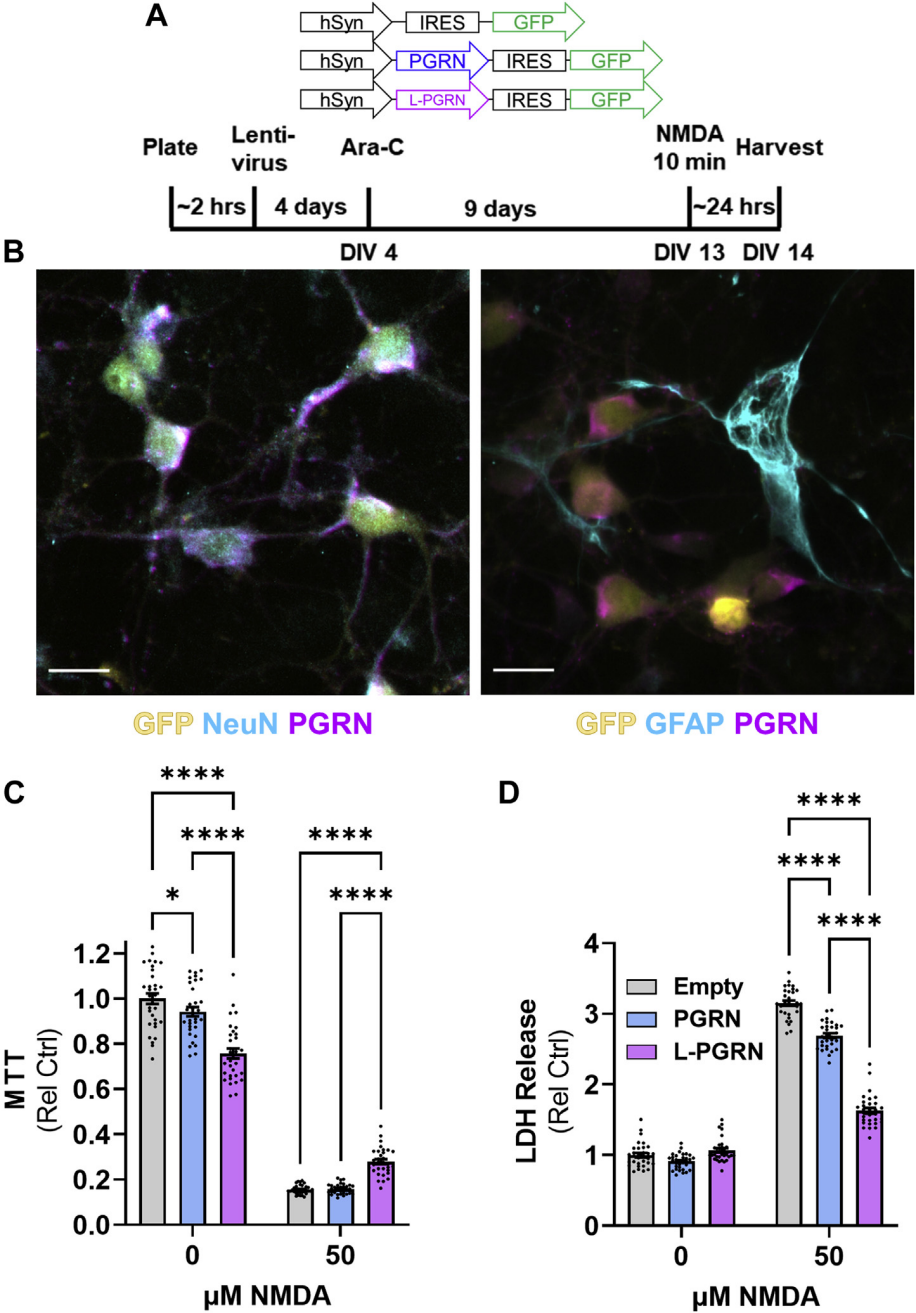
**Selective transduction of neurons with PGRN and L-PGRN protects against NMDA excitotoxicity.***A*, using the same design described in Figure 2, primary cortical neurons were transduced with lentiviral vectors expressing IRES-GFP under the synapsin promoter. An empty IRES-GFP vector was used as a control for PGRN-IRES-GFP and L-PGRN-IRES-GFP vectors. *B*, representative images of a cortical culture transduced with the hSyn-L-PGRN-IRES-GFP vector show selective transduction of neurons (scale bar = 20 μm). *C*, similar to results with PGK lentiviral vectors, transduction with L-PGRN protected against loss of viability in the MTT assay 24 h after treatment with 50 μM NMDA (ANOVA effect of NMDA, *p* < 0.0001, effect of vector, *p* = 0.0016, NMDA x vector interaction, *p* < 0.0001, n = 32 per group). *D*, both PGRN and L-PGRN protected against cell death as assessed by LDH release 24 h after treatment with 50 μM NMDA (ANOVA effect of NMDA, *p* < 0.0001, effect of vector, *p* < 0.0001, NMDA x vector interaction, *p* < 0.0001, n = 32 per group). For all analyses ∗*p* < 0.05 and ∗∗∗∗*p* < 0.0001 by Tukey’s post-hoc test.

**Figure 5:**
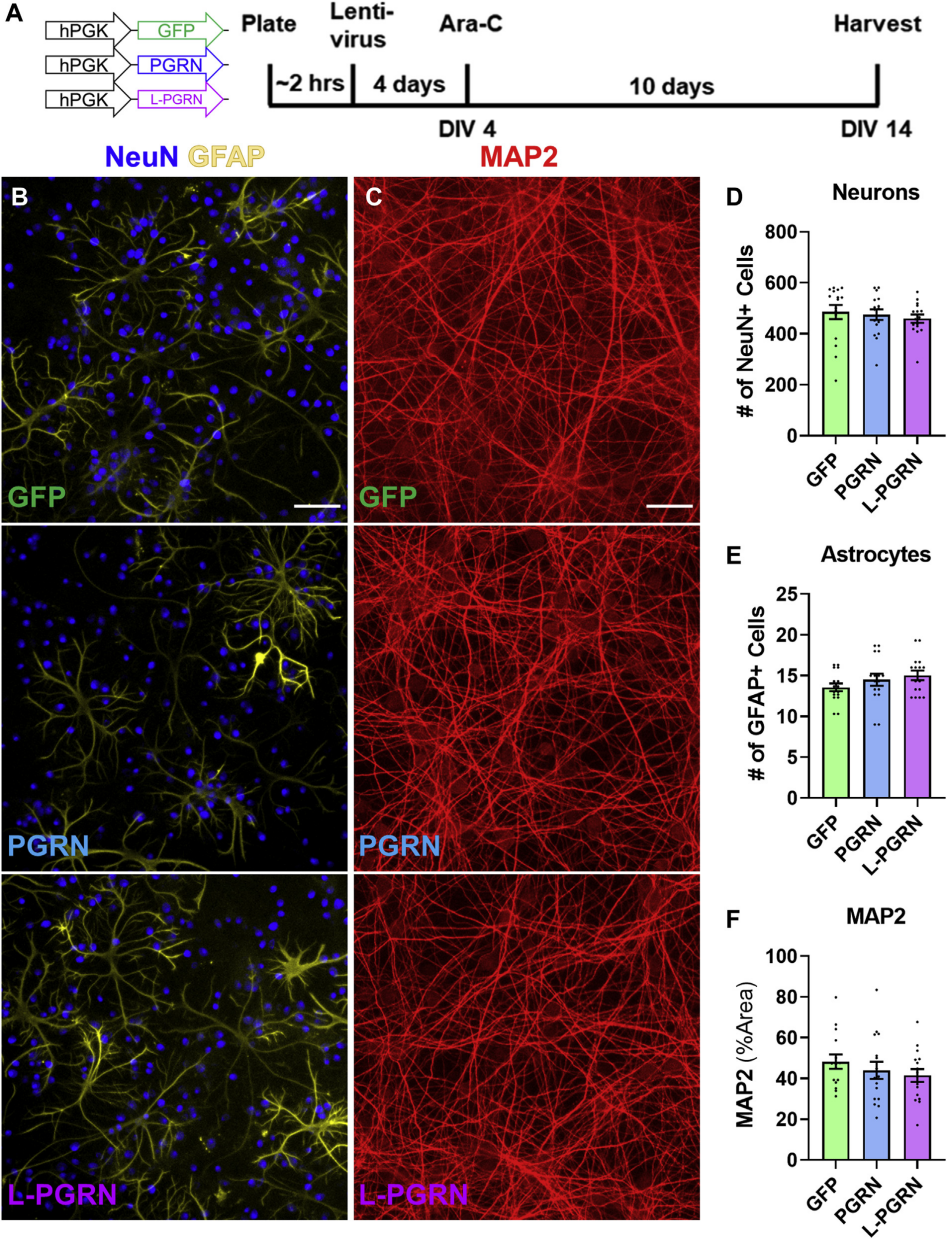
**Transduction with PGRN and L-PGRN does not alter the cellular composition of primary cortical cultures.***A*, primary neuronal cultures were transduced with vectors expressing GFP, PGRN, or L-PGRN under the PGK promoter shortly after plating, then analyzed at DIV14. *B* and *C*, cultures were immunostained for NeuN and GFAP to identify neurons and astrocytes, and for MAP2 to assess neuronal dendritic outgrowth. *D*–*F*, no significant differences in any of these markers were observed in cultures transduced with each vector (n = 15–16 per group). Scale bars in *B* and *C* represent 100 μm.

Based on our findings thus far, we hypothesized that L-PGRN’s protective effects were mediated through neuronal lysosomes. However, it might be possible that L-PGRN could act in astrocytic lysosomes to shift astrocytes toward a neuroprotective phenotype, as our experiments were conducted on mixed cultures of neurons and astrocytes (Figs. 4*B* and 5, *A*–*F*) using lentiviral vectors with a nonspecific promoter (PGK). To distinguish between these possibilities, we transduced cultures with lentiviral vectors using the neuron-specific synapsin (hSyn) promoter (Fig. 4, *A* and *B*). All vectors contained IRES-GFP, and an empty vector served as a control for the PGRN and L-PGRN vectors. In contrast to the PGK vectors, the hSyn PGRN and L-PGRN vectors modestly reduced baseline viability in the MTT assay (Fig. 4*C*). However, L-PGRN still preserved neuronal viability after NMDA treatment (Fig. 4*C*), and both PGRN and L-PGRN reduced neuronal death after NMDA treatment as assessed by LDH release (Fig. 4*D*). These data support the hypothesis that L-PGRN’s neuroprotective effects are mediated through neuronal lysosomes.

### Lysosome-targeted progranulin does not alter the cellular composition of primary cortical cultures

To investigate how progranulin might act in neuronal lysosomes to protect against excitotoxicity, we first assessed whether L-PGRN might alter the general properties of our primary cortical cultures. We assessed the effects of PGRN and L-PGRN on the cellular composition or our cultures by immunostaining for neuronal (NeuN, MAP2) and astrocytic (GFAP) markers (Fig. 5, *B* and *C*). We observed no significant change in numbers of neurons (Fig. 5*D*) or astrocytes (Fig. 5*E*) or the area of MAP2 labeling (Fig. 5*F*). We did not observe any cells positive for the microglial marker CD68 in our cultures.

### Lysosome-targeted progranulin does not alter neuronal activity and calcium influx after treatment with an excitotoxic dose of NMDA

**Figure 6:**
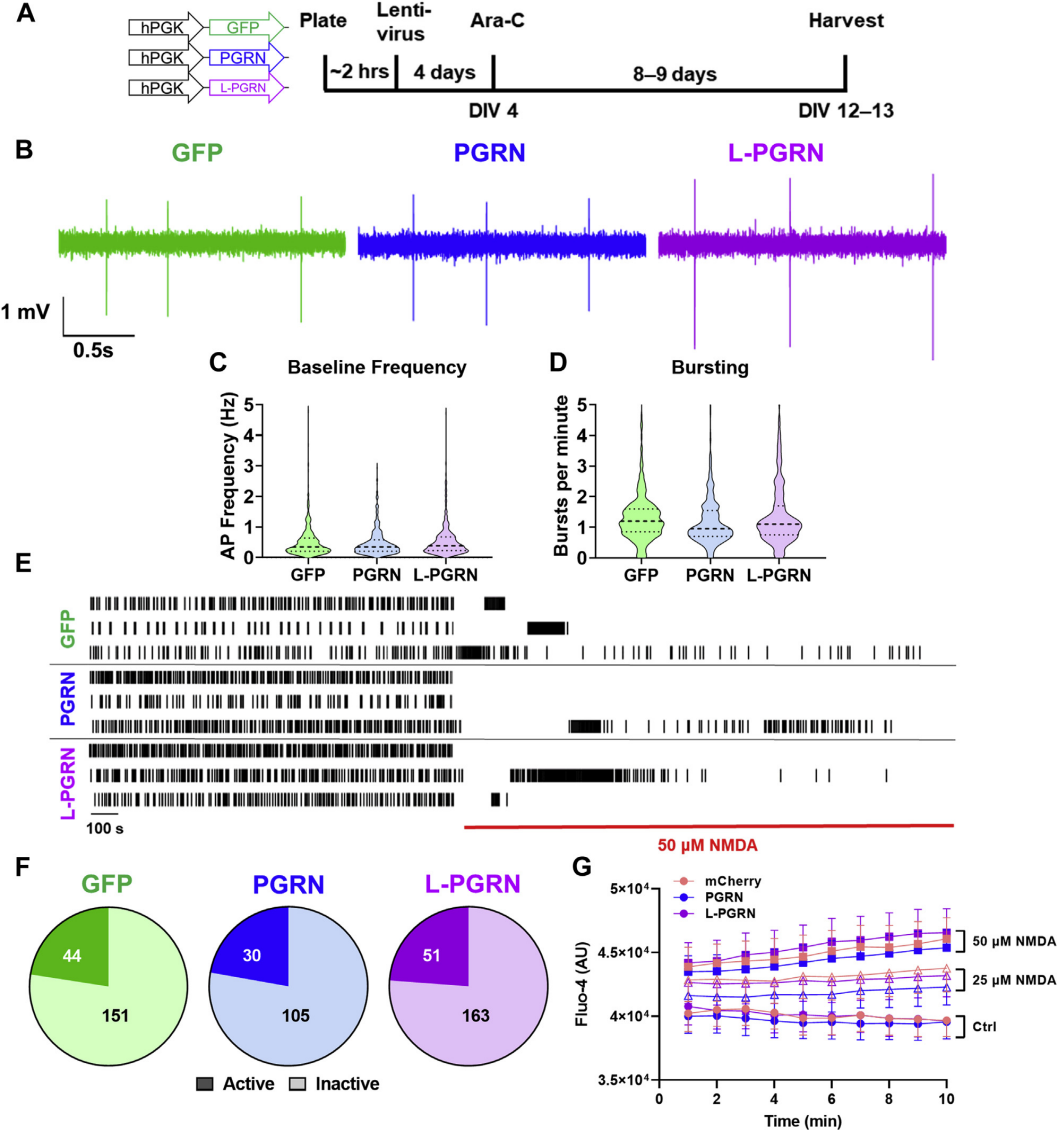
**Transduction with PGRN or L-PGRN does not alter baseline neuronal activity or the response to an excitotoxic dose of NMDA.***A*, primary neuronal cultures were transduced with vectors expressing GFP, PGRN, or L-PGRN under the PGK promoter shortly after plating, then analyzed at DIV12 to 13. *B*, neuronal network activity was assessed by multielectrode array, which revealed no differences in action potential frequency (*C*) or burst firing (*D*) between cultures transduced with each vector (n = 443–584 neurons per group). *E*, application of 50 μM NMDA for 10 min resulted in a dramatic decrease in firing, with most neurons becoming inactive (no action potentials within last 5 min of recording). *F*, the proportion of inactive neurons did not differ between lentiviral groups (Chi-square test, *p* = 0.9275, numbers on pie charts denote numbers of active or inactive neurons, n = 135–214 per group). *G*, all groups also exhibited a dose-dependent increase in intracellular calcium after NMDA treatment as assessed by fluorescence of the calcium-sensitive dye Fluo-4 (repeated measures ANOVA effect of NMDA *p* = 0.001, time x NMDA interaction *p* < 0.001, n = 6–11 per group), but this increase did not differ between lentiviral groups (repeated measures ANOVA effect of vector *p* = 0.732, vector x NMDA interaction, *p* = 0.997).

We also investigated whether L-PGRN might protect against excitotoxicity by altering neuronal firing or the physiologic response to NMDA. We assessed neuronal activity with multielectrode arrays (MEA) and found that neurons transduced with GFP, PGRN, and L-PGRN exhibited similar activity at baseline (Fig. 6, *B*–*D*). After treatment with excitotoxic doses of glutamate or NMDA, neurons cease firing action potentials due to persistent membrane depolarization (depolarization block) and exhibit sustained increases in intracellular calcium (68). L-PGRN did not alter the proportion of neurons exhibiting depolarization block (Fig. 6, *E* and *F*), nor did it alter the rise in intracellular calcium relative to mCherry-transduced neurons as assessed by the calcium-sensitive dye Fluo-4 AM (Fig. 6*G*). The protective effects of L-PGRN are therefore likely to be mediated downstream of the membrane depolarization and increased intracellular calcium caused by excitotoxic NMDA treatment.

### Lysosome-targeted progranulin blunts the autophagic response to excitotoxic doses of NMDA

**Figure 7:**
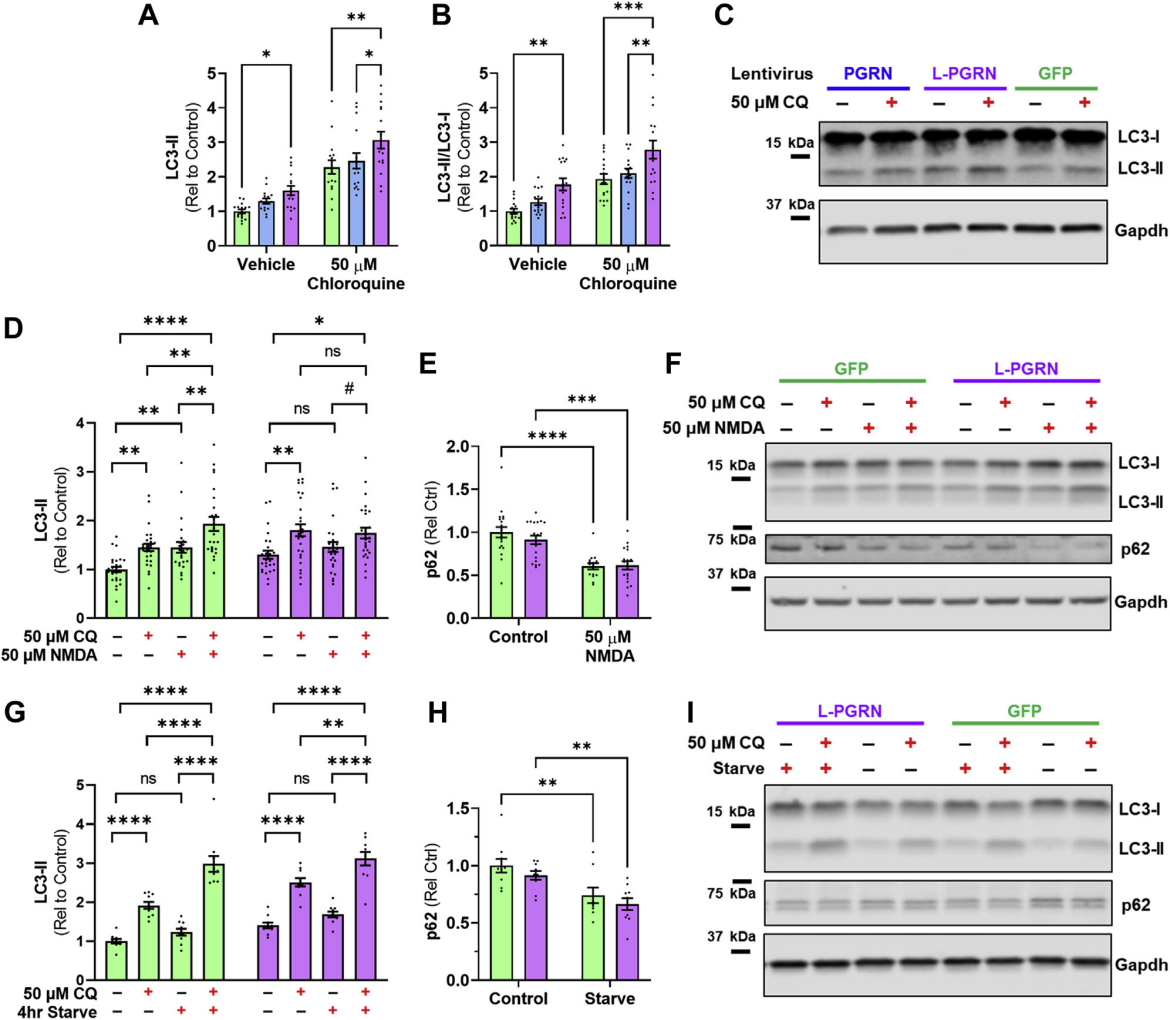
**L-PGRN blunts the autophagic response to an excitotoxic dose of NMDA.***A*–*C*, relative to GFP-transduced controls, neurons transduced with L-PGRN exhibited higher levels of LC3-II (*A*, ANOVA effect of vector *p* = 0.0004, effect of chloroquine *p* < 0.0001, ∗*p* < 0.05 and ∗∗*p* < 0.01 by Tukey’s post-hoc test. n = 16 per group) and LC3-II/LC3-I ratio (*B*, ANOVA effect of vector *p* < 0.0001, effect of chloroquine *p* < 0.0001, ∗∗*p* < 0.01 and ∗∗∗*p* < 0.001 by Tukey’s post-hoc test. n = 16 per group) both at baseline and after incubation with 50 μM chloroquine, consistent with a constitutive increase in autophagy. *D*–*F*, three hours after 50 μM NMDA treatment, neurons transduced with GFP or L-PGRN exhibited distinct changes in LC3-II (three-way ANOVA vector x NMDA interaction, *p* = 0.0042, n = 24–28 per group). NMDA increased LC3-II levels in GFP-transduced neurons (ANOVA effect of NMDA, *p* < 0.0001, ∗∗*p* < 0.01, ∗∗∗∗*p* < 0.0001 by Tukey’s post-hoc test), and the significant increase in LC3-II between neurons treated with 50 μM NMDA +50 μM chloroquine *versus* only 50 μM chloroquine confirmed an increase in autophagic flux. In contrast, NMDA failed to increase LC3-II in L-PGRN–transduced neurons (ANOVA effect of NMDA, *p* = 0.6323). However, chloroquine increased LC3-II both with and without addition of 50 μM NMDA (ANOVA effect of chloroquine, *p* = 0.0003, ^#^*p* < 0.1, ∗*p* < 0.05 by Tukey’s post-hoc test), indicating a lack of NMDA response rather than an impairment of autophagic flux. *E*, in contrast, NMDA decreased p62 in both GFP- and L-PGRN–transduced neurons (ANOVA effect of NMDA, *p* < 0.0001, ∗∗∗*p* < 0.001, ∗∗∗∗*p* < 0.0001 by Sidak’s post-hoc test). *G*–*I*, to determine if L-PGRN altered the autophagic response to other stimuli, we assessed the effects of 4 h of nutrient starvation on LC3-II and p62 levels. Nutrient starvation increased LC3-II levels in both groups, as confirmed both by global analysis (*I*, three-way ANOVA effect of starvation *p* < 0.0001, starvation x chloroquine interaction *p* < 0.0001, n = 10 per group) and subsequent analysis of each lentiviral group (GFP: ANOVA effect of starvation, *p* < 0.0001, effect of chloroquine, *p* < 0.0001, starvation x chloroquine interaction, *p* = 0.0016; L-PGRN: ANOVA effect of starvation, *p* = 0.0004, effect of chloroquine, *p* < 0.0001, ∗∗*p* < 0.01, ∗∗∗∗*p* < 0.0001 by Tukey’s post-hoc test). *H*, in further confirmation of autophagy induction, starvation reduced p62 levels among all neurons, but this was also similar among lentiviral groups (ANOVA effect of starvation *p* < 0.0001, ∗∗*p*< 0.01 by Sidak’s post-hoc test, n = 10 per group).

Autophagy promotes neuronal death in several models of excitotoxicity (69, 70, 71, 72, 73, 74) and progranulin is necessary for maintaining neuronal autophagy (21, 26). We therefore hypothesized that L-PGRN might reduce excitotoxic death by altering autophagy or the autophagic response to excitotoxic doses of NMDA. To begin testing this hypothesis, we first assessed the effects of PGRN and L-PGRN on autophagy in rat primary cortical neurons by measuring LC3-II levels at baseline and after incubation with 50 μM chloroquine (75). L-PGRN increased LC3-II levels under both conditions (Fig. 7, *A* and *B*), indicating an increased level of autophagy, which is consistent with a prior study of transgenic progranulin overexpression in mice (21). L-GFP failed to increase LC3-II relative to GFP (Fig. S1), suggesting that the L-PGRN’s stimulation of autophagy is a specific effect of lysosomal delivery of progranulin.

Previous studies have shown that excitotoxic doses of glutamate receptor agonists stimulate autophagy (76, 77), so we assessed the effect of L-PGRN on the autophagic response to 50 μM NMDA. Consistent with prior studies, we found that treatment with 50 μM NMDA increased LC3-II levels with a peak roughly 3 h post treatment (Fig. S2), consistent with a transient increase in autophagy. We therefore exposed GFP- and L-PGRN–transduced neurons to 50 μM NMDA for 3 h and assessed LC3-II levels both with and without addition of 50 μM chloroquine (75). NMDA increased LC3-II levels in GFP-transduced neurons, and this effect was further enhanced in the presence of chloroquine, indicating an increase in autophagic flux after NMDA treatment. (Fig. 7*D*). In contrast, NMDA did not increase LC3-II levels in L-PGRN–transduced neurons, either with or without chloroquine. This likely reflects failure of NMDA to stimulate autophagy rather than an impairment of autophagic flux, as chloroquine increased LC3-II levels in L-PGRN–transduced neurons regardless of the presence of NMDA (Fig. 7*D*).

We also assessed changes in p62 following NMDA treatment in GFP- and L-PGRN–transduced neurons and observed a significant decrease in both groups after NMDA treatment (Fig. 7*E*). L-PGRN may therefore not entirely block the autophagic response to NMDA in primary cortical neurons, though the LC3 data shows a significant blunting of this response.

To determine if L-PGRN specifically blocks the autophagic response to excitotoxicity or may block autophagy induction in response to other stimuli, we assessed whether L-PGRN affected the induction of autophagy by nutrient starvation, a classic autophagy-inducing stimulus (75). GFP- and L-PGRN–transduced neurons exhibited similar induction of autophagy after 4 h of nutrient starvation as shown by both an increase in LC3-II (Fig. 7*G*) and decrease in p62 (Fig. 7*H*). These data indicate that L-PGRN does not generally inhibit the induction of autophagy. The blunting of the autophagic response to NMDA may therefore reflect a more specific change to autophagy under excitotoxic conditions.

### L-PGRN occludes the protective effects of autophagy inhibition

**Figure 8:**
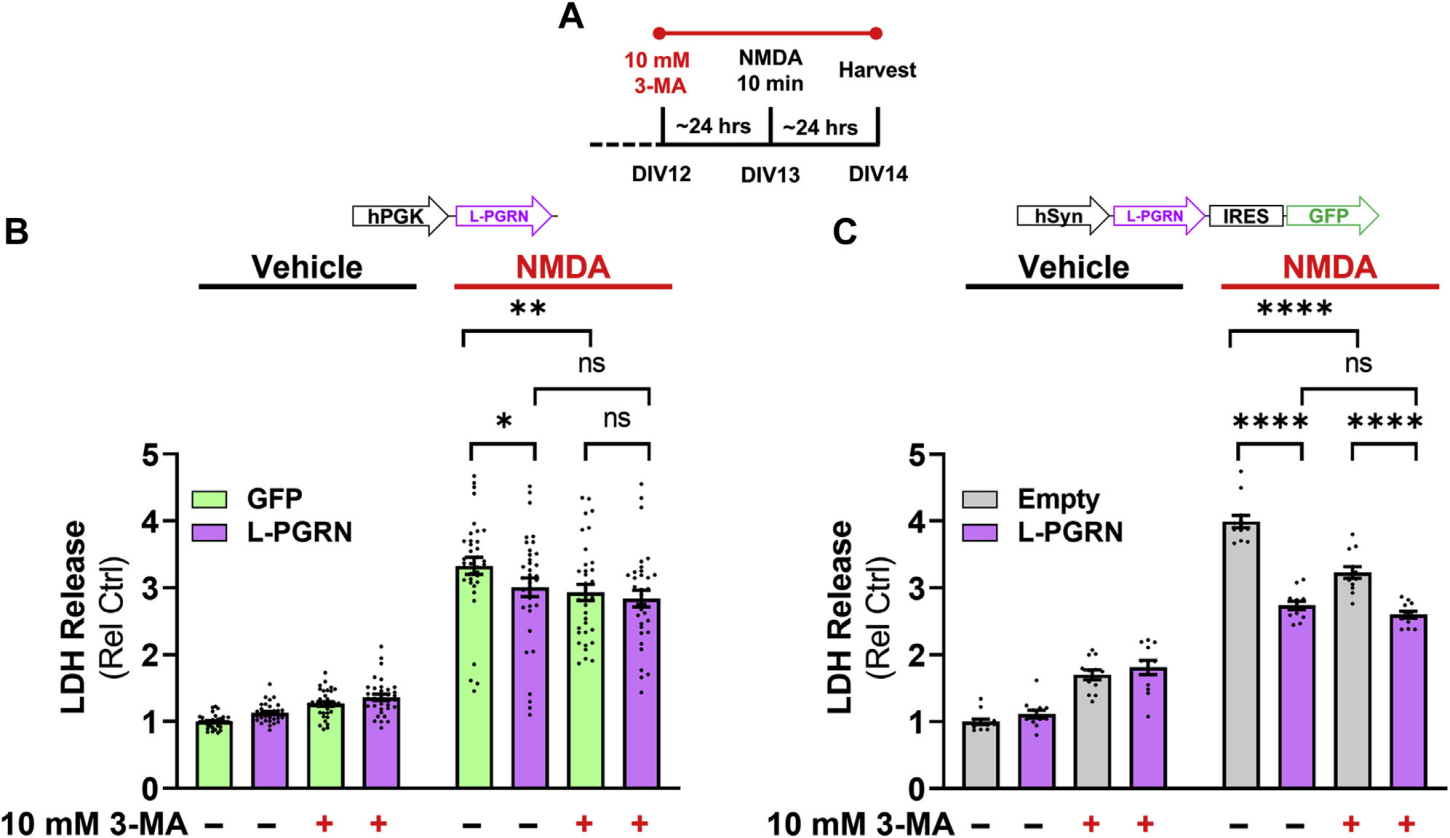
**L-PGRN occludes the protective effects of 3-MA.***A*, to assess the role of autophagy in the protective effects of L-PGRN, neurons transduced with GFP or L-PGRN PGK or hSyn lentiviral vectors were pretreated with vehicle or the autophagy inhibitor 3-methyladenine (3-MA, 10 mM) 24 h before NMDA treatment and assessment of cell death by LDH release. *B*, lenti-PGK-L-PGRN exerted the expected protective effect in the absence of 3-MA (three-way ANOVA vector x NMDA interaction, *p* = 0.0176, ∗*p* < 0.05 by Fisher’s LSD post-hoc test, n = 33–36 per group), and 3-MA treatment exerted a protective effect only in neurons transduced with GFP (three-way ANOVA 3-MA x NMDA interaction, *p* < 0.0001, ∗∗*p* < 0.01 by Fisher’s LSD post-hoc test). *C*, similarly, lenti-hSyn-L-PGRN exerted a protective effect in the absence of 3-MA (three-way ANOVA effect of vector, *p* < 0.0001, vector x NMDA interaction, *p* < 0.0001, ∗∗∗∗*p* < 0.0001 by Fisher’s LSD post-hoc test, n = 12 per group). 3-MA also exerted a protective effect that was selective for neurons treated with the empty vector (three-way ANOVA vector x 3-MA interaction, *p* = 0.0042, vector x 3-MA x NMDA interaction, *p* = 0.0035, ∗∗∗∗*p* < 0.0001 by Fisher’s LSD post-hoc test). In contrast to the PGK-L-PGRN lentiviral vector, lenti-hSyn-L-PGRN still reduced excitotoxicity in the presence of 3-MA, but this effect was attenuated, as 3-MA reduced LDH release in neurons transduced with the empty hSyn vector, but not the L-PGRN vector.

Prior studies have found that inhibiting autophagy with compounds such as 3-methyladenine (3-MA) protects against excitotoxic neuronal death (69, 70, 71, 73, 74). Since L-PGRN blunts the autophagic response to an excitotoxic dose of NMDA (Fig. 7*D*), we used 3-MA to determine if L-PGRN’s protective effects might be mediated through the autophagy-lysosomal pathway. Consistent with prior studies, we found that 3-MA treatment beginning 24 h before NMDA exposure (Fig. 8*A*) reduced cell death in neurons treated with either PGK-GFP or empty hSyn-IRES-GFP vectors (Fig. 8, *B* and *C*). In contrast, L-PGRN expressed by either vector occluded this protective effect, as 3-MA did not alter LDH release after NMDA treatment in L-PGRN–transduced neurons. Since 3-MA was protective only in GFP-transduced neurons, 3-MA masked the protective effect of PGK-L-PGRN relative to GFP and attenuated the protective effect of hSyn-L-PGRN-IRES-GFP relative to the empty IRES-GFP vector.

We also analyzed the effect of 3-MA in the MTT assay (Fig. S3), but interpretation of these data was complicated by an overall decrease in MTT signal in 3-MA–treated neurons. In experiments with both PGK and hSyn vectors, 3-MA treatment decreased MTT signal nearly as much as NMDA treatment, though LDH release (Fig. 8, *B* and *C*) and visual inspection of the cells showed that this was not due to cell death. We therefore suspect that 3-MA treatment may have had metabolic effects that reduced the baseline signal in the MTT assay. However, we still observed loss of MTT signal with NMDA treatment, and the protective effects of both PGK-L-PGRN and hSyn-L-PGRN-IRES-GFP were blocked by 3-MA treatment as was observed in the LDH assay (Fig. 8, *B* and *C*).

In summary, L-PGRN blunts the autophagic response to an excitotoxic dose of NMDA (Fig. 7*D*) and occludes the protective effect of autophagy inhibition with 3-MA (Fig. 8, *B* and *C*). Based on these data, we conclude that L-PGRN may protect against excitotoxicity by blocking the autophagic contribution to excitotoxic cell death.

## Discussion

The goal of this study was to investigate the mechanism underlying progranulin’s neuroprotective effects. As a secreted protein that is efficiently taken up and trafficked to lysosomes (42, 43, 45), progranulin has the ability to both signal through cell-surface receptors (27, 39) and to regulate aspects of autophagy-lysosomal function (22, 26, 46, 47, 48, 49, 50, 51, 52, 53, 78). To determine which of these functions might underlie progranulin’s neuroprotective effects, we developed a lentiviral vector that expressed lysosome-targeted progranulin (L-PGRN), which was designed to bypass progranulin secretion and deliver progranulin to lysosomes using the transmembrane domain and cytosolic tail of LAMP-1. L-PGRN did not undergo secretion at detectable levels, but retained the lysosomal localization and function of progranulin, as it was cleaved into granulins and normalized lysosomal enzyme activity in *Grn*^−/−^ neurons. L-PGRN was more consistently protective against NMDA excitotoxicity than PGRN, showing that extracellular progranulin is not necessary for progranulin’s protective effects against excitotoxicity. Instead, L-PGRN appears to act through neuronal lysosomes to block the contribution of autophagy to excitotoxic cell death.

These data provide insight into the mechanisms underlying progranulin’s neurotrophic effects. Progranulin overexpression or treatment with recombinant progranulin enhances neuronal survival (15, 16, 17) and growth (15, 18, 19, 20), improves recovery from injury (21, 22, 27), and protects neurons from various stressors (23, 24, 25). Previous data provided evidence that these effects might be mediated by either neurotrophic signaling (23, 24, 25, 27) or enhancement of autophagy-lysosomal function (21, 22). While our findings do not rule out a role for neurotrophic signaling in other effects of progranulin such as promoting axonal regrowth after injury (27), they do indicate that progranulin’s protective effects against excitotoxicity (17, 25) are likely to be mediated through lysosomes rather than by extracellular signaling.

This study also adds to the literature on the importance of the autophagy-lysosomal pathway in FTD due to progranulin mutations (FTD-*GRN*). Progranulin is critical for maintaining lysosomal function (5, 6, 7), and FTD-*GRN* patients show signs of lysosomal dysfunction, including accumulation of lysosomal proteins (64) and lysosomal storage material (79), abnormal lysosomal enzyme activity (48), and elevated levels of extracellular vesicles in plasma and brain tissue (80). Polymorphisms in the lysosomal gene *TMEM106B* modulate risk for FTD-*GRN*, showing that lysosomal changes modulate risk for FTD-*GRN* (81, 82, 83, 84). While progranulin’s specific function is unclear, it regulates multiple aspects of autophagy-lysosomal function, including activity of lysosomal enzymes (22, 46, 47, 48, 49, 50, 51, 52, 53), lysosomal pH (78), and autophagy (21, 26). This study, along with studies of progranulin overexpression in mice (21, 22), provides data associating progranulin’s lysosomal actions to its neurotrophic effects.

There is strong evidence that autophagy protects against neurodegenerative disease (85, 86), but autophagy appears to promote cell death in models of acute excitotoxicity (69, 70, 71, 73, 74). We replicated prior observations that inhibiting autophagy with 3-MA protects against excitotoxicity in neurons transduced with control lentiviral vectors. The blunting of the autophagic response to an excitotoxic dose of NMDA (Fig. 7*D*) and occlusion of 3-MA’s protective effects by L-PGRN (Fig. 8, *B* and *C*) suggests that L-PGRN blocks the autophagic contribution to excitotoxic cell death. Notably, this was not due to a general impairment in autophagy induction (Fig. 7, *G*–*I*) or autophagic flux (Fig. 7, *A*–*C*), as L-PGRN increased basal autophagy as defined by accumulation of LC3-II after treatment with a lysosomal inhibitor such as chloroquine (75).

Though the data do not provide strong support for this possibility, we cannot entirely rule out a role for extracellular signaling by granulins in protecting against excitotoxicity. L-PGRN did not produce detectable levels of progranulin, but did increase levels of extracellular granulins and multigranulin fragments (Figs. 1*F* and 3*A*). Prior work has shown that granulin E promotes neuronal survival (15, 54) over time courses ranging from 1 to 5 days after treatment (15, 54). We found that transfer of L-PGRN conditioned media to naïve neurons 1 or 24 h before NMDA treatment (1–2 days before assessment of viability/cell death) did not reproduce the protective effects of transduction with L-PGRN. This suggests that granulins do not protect against NMDA excitotoxicity by an acute neurotrophic signaling mechanism, but cannot exclude the possibility that long-term neurotrophic signaling by granulins may protect against NMDA.

An additional caveat to this study is that the LAMP-1 transmembrane/cytosolic tag represents an artificial method of delivering progranulin to lysosomes. Progranulin is normally delivered to lysosomes by sortilin (45) or by cotrafficking with prosaposin (42). A large portion of progranulin appears to be secreted (37, 38), then taken back up *via* these two pathways, though direct trafficking from the Golgi to the endolysosomal pathway may also occur (42). L-PGRN is likely to largely bypass these pathways and travel to lysosomes based on the sorting signal in LAMP-1’s cytosolic domain (87, 88). However, once in lysosomes, progranulin appears to be quickly cleaved into granulin fragments (43). Based on the presence of cathepsin L cleavage sites near progranulin’s C-terminus (60), we anticipate that once delivered to lysosomes, L-PGRN should be cleaved into granulin fragments that are indistinguishable from wild-type progranulin. Consistent with this prediction, we observed cleavage of both PGRN and L-PGRN into granulin fragments of uniform molecular weight (Fig. 1, *B* and *F*). We thus view L-PGRN as a useful tool to maintain progranulin’s lysosomal localization while bypassing its secretion.

The role of extracellular *versus* lysosomal progranulin in inflammation will be an important area for future investigation. Mouse models strongly implicate microglia in pathogenesis of FTD-*GRN* (29, 33, 34, 36, 89). *Grn*^−/−^ macrophages and microglia exhibit phenotypes with straightforward ties to lysosomal function such as impaired phagocytosis, pathogen clearance, and antigen presentation (26, 32, 36, 90). However, progranulin might also antagonize TNF receptors ((40) but also see (41)), and progranulin/granulins may act as signaling molecules to regulate immune responses to injury (30). Progranulin may therefore act *via* multiple mechanisms in regulating inflammation.

These data highlight lysosomes as a key site of action for progranulin’s neuroprotective effects and add to the literature showing that progranulin improves neuronal health by promoting autophagy-lysosomal function (21, 22, 26). This may provide insight into the protective effects of progranulin after injury (17, 21, 22, 27). Various types of injury stimulate progranulin expression and secretion by glial cells, especially microglia (17, 22, 27, 31, 91, 92, 93), that might deliver more progranulin to neuronal lysosomes, protecting against cell death and promoting recovery from injury. As such, the role of extracellular progranulin in interaction between cell types in the brain will be another important area for future investigation.

## Experimental procedures

### 293T cells

HEK293T cells were purchased from American Type Culture Collection (#CRL-3216) and grown in DMEM (Corning) with 10% FBS (Biotechne) and 1% penicillin/streptomycin (ThermoFisher). For initial vector characterization, 293T cells were transduced with lentiviral vectors at a multiplicity of infection (MOI) of 1000. Conditioned media and cell lysates were harvested 2 to 3 days later.

### Primary cortical neurons

All animal studies were approved by the Institutional Animal Care and Use Committee of the University of Alabama at Birmingham. E19 embryos were harvested from timed-pregnant Sprague Dawley rats obtained from Charles River. Cortical tissue was dissected in cold Hibernate E media (ThermoFisher), then digested with papain (Worthington Biochemical Corporation). For viability, cell death, or biochemical assays, neurons were plated into 24- or 48-well plates in Neurobasal medium (ThermoFisher) supplemented with B27 Plus (ThermoFisher), 2 mM L-Glutamine (ThermoFisher), and 10% FBS (BioTechne) at a density of 250,000 cells/well for 24-well plates (1.3 × 10^5^ cells/cm^2^) or 125,000 cells/well (1.1 × 10^5^ cells/cm^2^) for 48-well plates. For imaging, neurons were plated onto No. 1.5 glass coverslips (Electron Microscopy Sciences) at a density of 200,000 cells/well (1.3 × 10^5^ cells/cm^2^). Plates were coated overnight with 0.1 mg/ml poly-D-lysine (MilliporeSigma) and coverslips were coated with 0.67 mg/ml poly-D-lysine prior to use. Lentiviral transduction was conducted approximately 2 h after plating. Cultures not used for lentiviral transduction were subjected to a 75% media change with Neurobasal medium with B27 Plus and L-Glutamine, but no FBS. A 50% media change containing Cytosine β-D-arabinofuranoside (5 μM final concentration, MilliporeSigma) was conducted on DIV4 to limit glial growth. A second 50% media change was conducted at DIV9, and experiments were conducted on DIV12 to 14. Neurons were maintained at 37 °C and 5% CO2.

Mouse primary cortical neurons were obtained by breeding *Grn*^*+/+*^ or *Grn*^−/−^ mice (33) to mates of the same genotype. Embryos were harvested at E15 and cultured as described above. Experiments were conducted on cultures of *Grn*^*+/+*^ and *Grn*^−/−^ neurons run in parallel.

### Lentiviral plasmids

Lentiviral vectors were constructed by cloning into the vector pRRLSIN.cPPT.PGK-GFP.WPRE (Addgene plasmid # 12252; http://n2t.net/addgene:12252; RRID:Addgene_12252), which was a gift from Didier Trono. We constructed pRRL-HA-GRN (PGRN) by inserting an HA tag after the signal peptide of human *GRN*, then cloning the resulting fragment into the pRRL vector using In-Fusion (Takara Bio). pRRL-HA-GRN-LAMP1 (L-PGRN) was constructed using PCR to add the sequence of the transmembrane domain and cytosolic tail of human LAMP-1 (62) to HA-GRN, then cloning the resulting fragment into the pRRL vector with In-Fusion (Takara Bio). For both vectors, pRRL was cut with BamHI and SalI (New England Biolabs) to enable insertion of the GRN or GRN-LAMP fragments.

For neuron-specific targeting, we cloned an IRES-GFP site (94) into the backbone of the lentiviral vector lenti SYN-FLAG-dCas9-VPR (Addgene plasmid # 114196; RRID:Addgene_114196, a gift from Jeremy Day) (95) using AgeI and EcoRI (New England Biolabs). This empty vector served as the control hSyn vector. PGRN and L-PGRN were inserted before the IRES site to generate hSyn-PGRN and hSyn-L-PGRN vectors.

### Lentiviral vector packaging

Lentiviral vectors were produced by adapting previously described methods (95, 96). HEK293T Cells (ATCC, #CRL-3216) were seeded in T75 or T225 flasks and cotransfected using Fugene HD (Promega) with one of the lentiviral transfer vectors described above, as well as the envelope plasmid pMD2.G (Addgene plasmid # 12259; http://n2t.net/addgene:12259; RRID:Addgene_12259) and the second-generation packaging plasmid psPAX2 (Addgene plasmid # 12260; http://n2t.net/addgene:12260; RRID:Addgene_12260), both of which were gifts from Didier Trono. Approximately 48 h after transfection, media was collected, centrifuged for 5 min at 2300*g*, and filtered through a 0.45 μm syringe filter. The clarified media was then ultracentrifuged at 25,000 rpm on either SW-41 or SW-32 rotors (Beckman Coulter) for 105 min. The resulting pellet was resuspended in sterile PBS to generate a concentrated lentiviral preparation. Lentiviral titer was determined using the Lenti-X qRT-PCR titration kit (Takara Bio).

### Lysosome immunoprecipitation

Lysosomes were enriched using Tmem192 immunoprecipitation (63). 293T cells were cotransfected with pRRL-GFP, PGRN, or L-PGRN vectors and a Tmem192-2x Flag vector (Addgene plasmid # 102929; RRID:Addgene_102929, a gift from David Sabatini) (63) in 6-well plates using OMNIfect transfection reagent (Transomic Technologies). Forty-eight hours after transfection, cells were scraped into D-PBS, lysed with 20 strokes of a dounce homogenizer, and centrifuged at 1000*g* for 2 min. The supernatant was precleared with 20 μl of protein G dynabeads (ThermoFisher) for 10 min at room temperature. The precleared supernatant was then incubated with 5 μg of anti-Flag antibody (MilliporeSigma #F1804) bound to 50 μl of protein G dynabeads for 15 min at room temperature. The beads were pulled down and washed three times with D-PBS. Proteins were eluted by addition of 1× Laemmli buffer and heating at 95 °C for 5 min. Samples were immediately run on tris-glycine gels (Bio-Rad) for Western blot.

### Neuronal transduction and cell viability assays

All cell viability assays followed a similar experimental design. Approximately 2 h after plating, neurons were transduced with GFP, PGRN, or L-PGRN vectors at an MOI of 1000 (96). Lentiviruses were removed after overnight incubation (roughly 15 h) and replaced with 1:1 conditioned media and fresh Neurobasal+B27 Plus. Neurons were treated with NMDA (Tocris Biosciences) for 10 min at DIV13, which was then replaced with 1:1 conditioned media and fresh Neurobasal+B27 Plus. Cell viability was assessed 24 h later, on DIV14, using the assays described below. 3-MA (Acros Organics or Tocris Biosciences) was applied on DIV12, roughly 24 h before NMDA treatment.

### MTT assay

Cell viability was assessed by conversion of 3-(4,5-Dimethyl-2-thiazolyl)-2,5-diphenyl-2H-tetrazolium bromide (MTT) to a purple formazan product. MTT (MilliporeSigma) was added to neurons growing in 24-well plates at a final concentration of 83 to 100 μM. Neurons were then incubated at 37 °C for 30 min, then media was removed, and neurons were lysed with 300 μl DMSO (Fisher Scientific) per well. In total, 200 μl from each well was added to 96-well plates and absorbance was read at 590 nm on a Biotek Synergy LX plate reader.

### Calcein assay

Primary neuronal cultures were incubated with 3 μM Calcein AM (ThermoFisher) in DPBS for 30 min at 37 °C, then washed once with warm DPBS. Fluorescence was read on a Biotek Synergy LX plate reader.

### LDH assay

Conditioned media was collected from primary neuronal cultures, then placed on ice until analysis of LDH activity. Media was diluted in assay buffer (0.1 M potassium phosphate) containing 140 μM β-nicotinamide adenine dinucleotide and 1.9 μM sodium pyruvate. Absorbance was read at 340 nm on a Biotek Synergy LX plate reader every 15 s for 3 min.

### Propidium iodide staining

Media was aspirated from neuronal cultures and replaced with propidium iodide (50 μg/ml) (Thermo Fisher) in ice-cold DPBS. The cultures were incubated on ice for 5 min, then washed with DPBS, and immediately read on a Biotek Synergy LX plate reader.

### Western blot

Cells were harvested in lysis buffer (50 mM Tris, 150 mM NaCl, 5 mM ethylenediaminetetraacetic acid, 0.1% sodium dodecyl sulfate (SDS), 0.1% Triton-X-100, and 0.5% sodium deoxycholate) containing protease and phosphatase inhibitors (Halt protease inhibitor cocktail and phosphatase inhibitor cocktail, Thermo Fisher). Protein concentration was determined by BCA assay (Thermo Fisher). Uniform amounts of protein per sample were run on 10 or 15% polyacrylamide gels (Bio-Rad) and transferred to Immobilon-FL PVDF membranes (MilliporeSigma). For analysis of granulins, samples were transferred onto low-fluorescence PVDF membranes with 0.2 μm pores (Thermo Fisher). Membranes were blocked with protein-free blocking buffer (Thermo Fisher), then probed overnight with primary antibodies. The following day, membranes were probed with species-matched IRdye-conjugated secondary antibodies (Li-COR Biosciences) and scanned on an Odyssey scanner (Li-COR Biosciences). Blots of cell lysates were probed for Gapdh to confirm equal protein loading. Blots of conditioned media were conducted volumetrically (equivalent amounts of media loaded per lane). As neurons were plated at equal density and we detected no significant differences in cell number (Fig. 5), no additional loading control was analyzed for blots of conditioned media.

### Immunostaining

All cells were fixed for 30 min in 4% paraformaldehyde and 4% sucrose in PBS. For PGRN/LAMP-2 staining, cells were permeabilized with 0.5% saponin (MilliporeSigma), blocked in 3% BSA (MilliporeSigma), then incubated in primary antibody overnight in 3% BSA. For staining of cell types, cells were blocked in 5% normal goat serum, 5% donor horse serum, and 1% BSA with 0.5% saponin, then incubated overnight with primary antibody in the same buffer. The following day, cells were washed with PBS, then incubated with species-matched AlexaFluor-conjugated secondary antibodies (Thermo Fisher) using the same buffers as with primary antibodies. Cells were then washed with PBS and coverslips were mounted onto slides using Vectashield Hardset mounting medium with DAPI (Vector Laboratories). PGRN/LAMP-2 staining was imaged with a Nikon A1R inverted confocal microscope, and cell type immunostaining was imaged with a Thermo Fisher EVOS M5000 imaging system.

### Lysosomal enzyme activity assays

Enzyme activity assays were conducted as previously described (48) using the following 4-methylumbelliferone (4-MU)-conjugated substrates, both of which were purchased from MilliporeSigma: 4-MU-N-acetyl-β-D-glucosaminide (β-hexosaminidase) and 4-MU-β-D-glucuronide hydrate (β-glucuronidase). Lysates from primary cortical cultures were harvested in lysis buffer (50 mM Tris, 150 mM NaCl, 5 mM EDTA, 1% Triton X-100, 0.1% sodium deoxycholate) and incubated with substrates for 1 h at 37 °C in pH 4.2 citrate buffer, then stopped by addition of glycine buffer. Fluorescence was read on a Biotek Synergy LX plate reader at 360 nm excitation and 440 nm emission. Results were quantitated using a 4-MU standard curve run on each plate and are reported normalized to control values.

### Multielectrode array (MEA)

Neurons were plated on 48-well MEA plates at 30,000 cells per well in Neurobasal medium with B27 Plus, 2 mM L-Glutamine, and 10% FBS, and transduced with GFP, PGRN, or L-PGRN lentiviral vectors as described above. BrainPhys media (Stem Cell Technologies) was used instead of Neurobasal for media changes on DIV4 (with addition of Ara-C) and DIV9. At DIV 12, neurons were recorded on an Axion system as described previously (97). Neurons were recorded for 20 min for a baseline recording, then 50 μM NMDA was applied, and neuronal firing was recorded for 20 more minutes. Electrical activity was analyzed with Plexon Offline Sorter and NeuroExplorer to determine action potential frequency as described previously (97). Neurons were defined to be in depolarization block if there were zero action potentials in the last 5 min of recording after NMDA application.

### Fluo-4 fluorescence

Neurons were incubated with 4 μM Fluo-4 AM (Thermo Fisher) in Neurobasal medium for 30 min at 37 °C, then returned to Neurobasal medium for another 30 min before imaging. Neurons were treated with 0, 25, or 50 μM NMDA, then placed into a Biotek Synergy 2 plate reader held at 37 °C. Fluorescence was measured every minute for 10 min at 485 nm excitation and 528 nm emission.

### Lysosome labeling with DQ-BSA

To label lysosomes by unquenching of DQ-BSA fluorescence, primary cortical neurons were incubated overnight with DQ-Red BSA (Thermo Fisher, 5 μg/ml). The following morning, neurons were incubated for 2 h with fresh neurobasal medium, then fixed for immunostaining in 4% paraformaldehyde with 4% sucrose in PBS.

### Antibodies

The following antibodies were used for Western blot: Progranulin (R&D Systems #AF2420, 1:500 and MilliporeSigma #HPA008763, 1:500), NeuN (MilliporeSigma #MAB377, 1:500), GFAP (Agilent #Z033429, 1:500), LC3B (MilliporeSigma #L7543, 1:500), p62 (Proteintech #18420-1-AP, 1:1000), LAMP-1 (Santa Cruz Biotechnology #sc-20011, 1:1000), GM130 (Cell Signaling Technologies #12480, 1:1000), Grp94 (Santa Cruz Biotechnology #sc-32249, 1:500), Cytochrome C (Santa Cruz Biotechnology #sc-13156, 1:500), and Gapdh (MilliporeSigma #MAB374, 1:5000). Antibodies used for immunostaining included: Progranulin (R&D Systems #AF2420, 1:500), LAMP-2 (Invitrogen # PA1655, 1:500), GFP (Cell Signaling Technologies #2956, 1:500), Cathepsin D (R&D Systems #AF1029, 1:500), NeuN (MilliporeSigma #MAB377, 1:500), GFAP (Agilent #Z033429, 1:1000), and MAP2 (Invitrogen # PA110005).

### Statistics

Statistical analyses and sample sizes are described in each figure legend. Except where noted, data were analyzed by ANOVA with factors of lentiviral vector and, where applicable, NMDA treatment, chloroquine treatment, 3-MA treatment, or nutrient starvation. For three-way ANOVA of LC3-II after NMDA treatment or nutrient starvation, significant effects of vector or interaction of vector with other factors were followed by subsequent two-way ANOVA of each vector with factors of chloroquine and NMDA or starvation. Three-way ANOVA of the effects of 3-MA and NMDA on neuronal cell death was followed by a limited number of preplanned comparisons using Fisher’s LSD post-hoc test. For two-way ANOVA, significant main effects or interactions were followed by post-hoc analysis as described in the figure legends. The proportion of active neurons in MEA analysis was analyzed by Chi-square test. All analyses were conducted with GraphPad Prism 9 except for repeated measures ANOVA of Fluo-4 data, which was conducted with IBM SPSS 27. Two-tailed *p* values were calculated for all analyses, with α set at 0.05. Data are shown as mean ± SEM.

## Data availability

All data are contained in the figures, figure legends, or supporting information.

## Supporting information

This article contains supporting information.

## Conflict of interest

The authors declare that they have no conflicts of interest with the contents of this article.
