# Supplementary material for: Delivering progranulin to neuronal lysosomes protects against excitotoxicity
Source: J Biol Chem. 2021 Jul 21;297(3):100993. doi: 10.1016/j.jbc.2021.100993 (PMC8379502; doi:10.1016/j.jbc.2021.100993)
Supplement: Supplemental Figure S1 [file mmc1.docx]

**
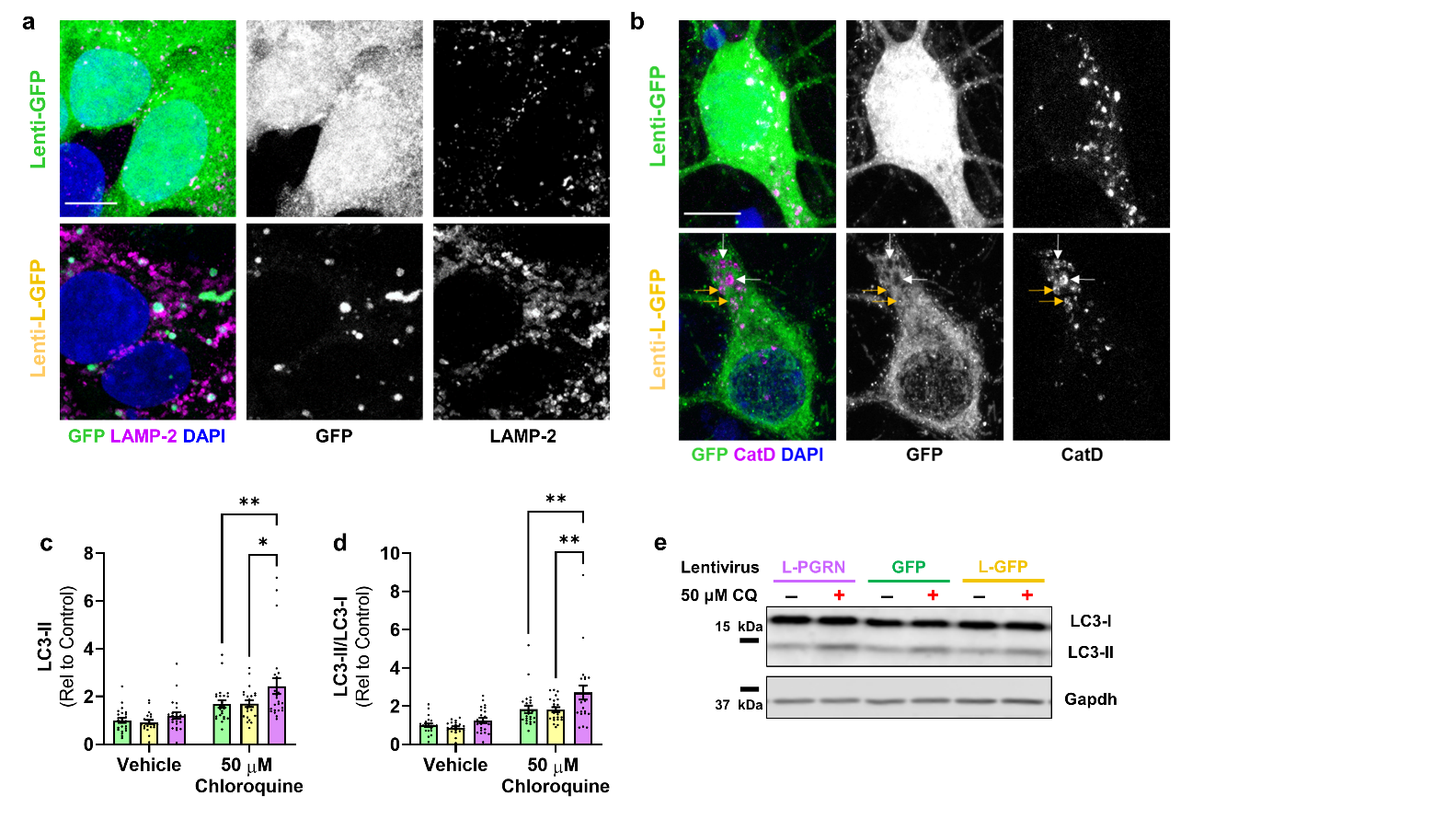
**

**Figure S1 – L-GFP localizes to lysosomes and appears to undergo lysosomal degradation, but does not stimulate autophagy.**

**a**, In HEK293T cells, transduction with lenti-PGK-GFP produced diffuse GFP fluorescence that filled the cell, but transduction with lenti-PGK-L-GFP produced punctate GFP fluorescence that primarily localized to LAMP-2–positive vesicles. The overall level of L-GFP fluorescence was lower than GFP, consistent with lysosomal degradation of L-GFP. **b**, Similarly, L-GFP produced much lower levels of fluorescence than GFP in primary cortical neurons. Unlike 293T cells, low levels of GFP immunoreactivity were observed throughout the cytoplasm of L-GFP transduced neurons. Many lysosomes (identified as cathepsin D (CatD)-positive vesicles) were devoid of any GFP immunoreactivity (white arrows), perhaps consistent with degradation of L-GFP. However, some lysosomes did exhibit GFP immunoreactivity (orange arrows). **c–e**, Unlike L-PGRN, L-GFP did not stimulate autophagy in primary cortical neurons. L-PGRN–transduced neurons exhibited higher levels of LC3-II (**c**, ANOVA effect of vector, *p* = 0.0088, * = *p* < 0.05, ** = *p* < 0.01 by Tukey’s post-hoc test, n = 22–24 per group) and LC3-II/LC3-I ratio (**d**, ANOVA effect of vector, *p* = 0.0016, ** = *p* < 0.01 by Tukey’s post-hoc test) after 4 hours of incubation with 50 μM chloroquine than both GFP- and L-GFP–transduced neurons. L-GFP–transduced neurons did not differ from GFP controls. Scale bars in **a** and **b** represent 10 μm. Images in **a** show native GFP fluorescence, while images in **b** show GFP immunoreactivity.
