# Supplementary material for: Delivering progranulin to neuronal lysosomes protects against excitotoxicity
Source: J Biol Chem. 2021 Jul 21;297(3):100993. doi: 10.1016/j.jbc.2021.100993 (PMC8379502; doi:10.1016/j.jbc.2021.100993)
Supplement: Supplemental Figure S2 [file mmc2.docx]

**
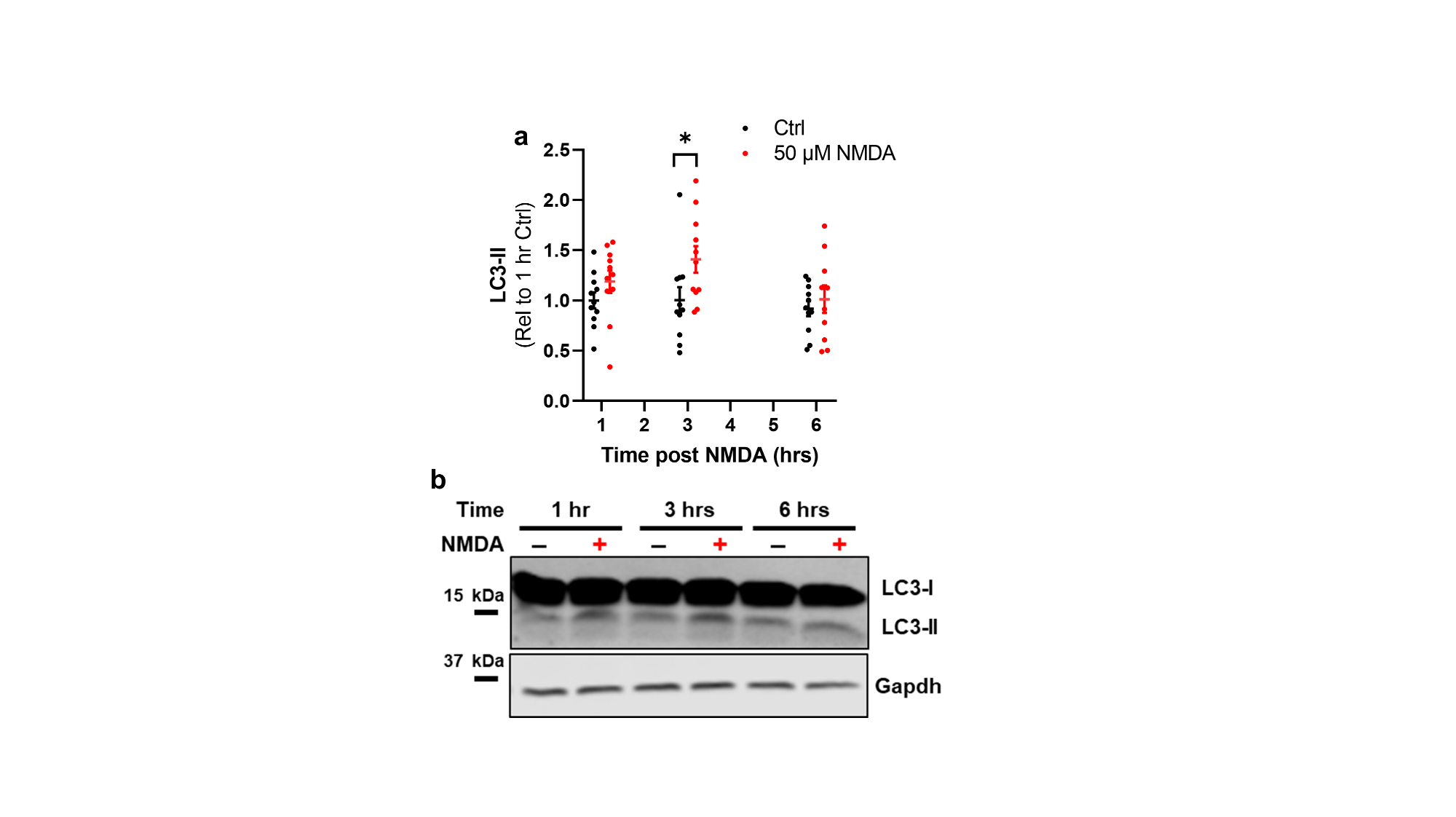
**

**Figure S2 – 50 μM NMDA Induces a Transient Increase in LC3-II.**

**a**, **b**, Treatment with 50 μM NMDA for 10 minutes induced a delayed increase in LC3-II levels that peaked around 3 hours post treatment and returned to baseline by 6 hours post treatment, consistent with a transient increase in autophagy (ANOVA effect of NMDA, *p* = 0.0159, * = *p* < 0.05 by Sidak’s post-hoc test, n = 10–11 per group).
