# Supplementary material for: Delivering progranulin to neuronal lysosomes protects against excitotoxicity
Source: J Biol Chem. 2021 Jul 21;297(3):100993. doi: 10.1016/j.jbc.2021.100993 (PMC8379502; doi:10.1016/j.jbc.2021.100993)
Supplement: Supplemental Figure S3 [file mmc3.docx]

**
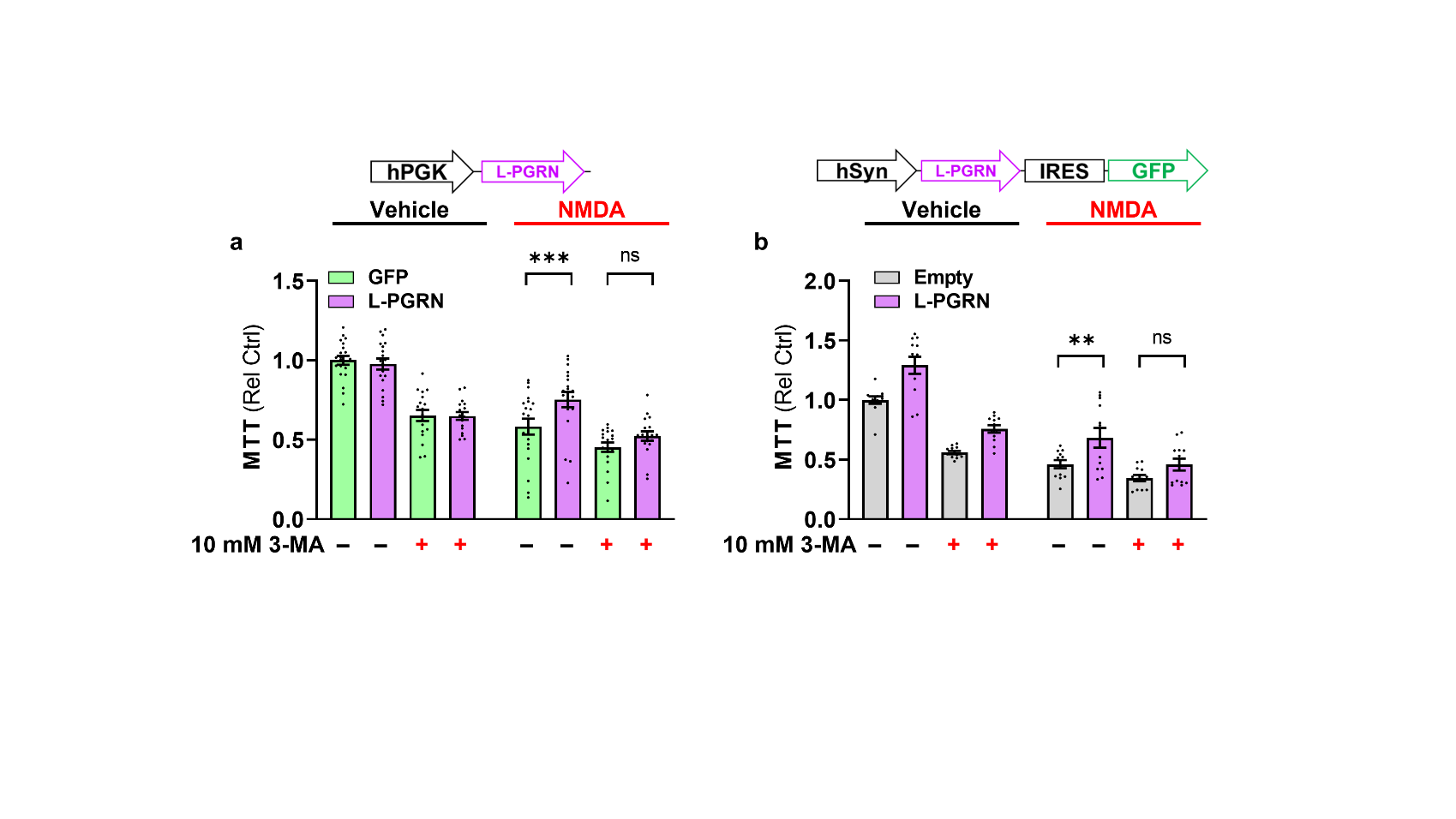
**

**Figure S3 – 3-MA Reduces MTT Signal and Masks the Protective Effect of L-PGRN.**

3-MA treatment reduced signal in the MTT assay in experiments with both PGK lentiviral vectors (**a**, 3-way ANOVA effect of 3-MA, *p* < 0.0001, n = 17–20 per group) and hSyn lentiviral vectors (**b**, 3-way ANOVA effect of 3-MA, *p* < 0.0001, n = 12 per group). We were nonetheless able to observe a decrease in MTT with NMDA treatment in both experiments (**a**, ANOVA effect of NMDA, *p* < 0.0001, n = 17–20 per group, **b**, 3-way ANOVA effect of NMDA, *p* < 0.0001, n = 12 per group). Both lenti PGK-L-PGRN (**a**, 3-way ANOVA effect of vector, *p* = 0.0403, vector x NMDA interaction, *p* = 0.0105, *** = *p* < 0.001 by Fisher’s LSD post-hoc test) and lenti-hSyn-L-PGRN (**b**, 3-way ANOVA effect of vector, *p* < 0.0001, ** = *p* < 0.01 by Fisher’s LSD post-hoc test) protected against NMDA in neurons not treated with 3-MA, but 3-MA occluded this effect for both vectors.
